# Discovery of a novel powdery mildew (*Blumeria graminis*) resistance locus in rye (*Secale cereale* L.)

**DOI:** 10.1038/s41598-021-02488-5

**Published:** 2021-11-29

**Authors:** N. M. Vendelbo, K. Mahmood, P. Sarup, P. S. Kristensen, J. Orabi, A. Jahoor

**Affiliations:** 1 Nordic Seed A/S, Odder, Denmark; 2 Department of Agroecology, Faculty of Technical Sciences, Aarhus University, Slagelse, Denmark; 3 Department of Plant Breeding, The Swedish University of Agricultural Sciences, Alnarp, Sweden

**Keywords:** Plant breeding, Plant breeding, Pattern recognition receptors in plants, Genome-wide association studies

## Abstract

Powdery mildew is one of the most destructive diseases in the world, causing substantial grain yield losses and quality reduction in cereal crops. At present 23 powdery mildew resistance genes have been identified in rye, of which the majority are in wheat-rye translocation lines developed for wheat improvement. Here, we investigated the genetics underlying powdery mildew resistance in the Gülzow-type elite hybrid rye (*Secale cereale* L.) breeding germplasm. In total, 180 inbred breeding lines were genotyped using the state-of-the-art 600 K SNP array and phenotyped for infection type against three distinct field populations of *B. graminis* f. sp. *secalis* from Northern Germany (2013 and 2018) and Denmark (2020). We observed a moderate level of powdery mildew resistance in the non-restorer germplasm population, and by performing a genome-wide association study using 261,406 informative SNP markers, we identified a powdery mildew resistance locus, provisionally denoted *PmNOS1,* on the distal tip of chromosome arm 7RL. Using recent advances in rye genomic resources, we investigated whether nucleotide-binding leucine-rich repeat genes residing in the identified 17 Mbp block associated with *PmNOS1* on recent reference genomes resembled known *Pm* genes.

## Introduction

Powdery mildew (PM) is one of the most devasting diseases globally^4^. In cereals, the causative agent of PM is the ascomycete fungus *Blumeria graminis* (DC.) speer (*Bg*), which is capable of inflicting severe grain yield loss (≥ 20%) and quality reduction in cereals^5–8^. In periods of conducive conditions, such as frequent precipitation and low to moderate temperatures, *Bg* can cause severe epidemics by repeated infections and clonal reproduction^9^. As an obligate biotroph, *Bg* is highly specialized to its host species, divided into several distinct *‘formae specialis’* (f. sp.)^10^, and dependent on a living host for survival and reproduction. In the later stages of the growing season, or in periods of unconducive conditions, chasmothecia structures are formed^11^. The chasmothecia field inoculum facilitates the infection of volunteer plants or the successive autumn sown winter crop, allowing *Bg* to overwinter as dormant mycelia^12^. Long-distance dispersal by wind of conidiospores drives the large spatial variability in the *Bg* population^13–15^. In barley (*Hordeum vulgare* L.), the virulence complexity of *B. graminis* f. sp. *hordei* has been observed to increase with the prevailing wind direction from west to east in Europe^16^. Rather than geographical origin, evidence suggests that local use of host resistance and fungicides are the primary factors influencing the European *B. graminis* f. sp. *hordei* population^17^. These findings underline the seriousness of long-distance dispersal of *B. graminis* spores, which allows novel and aggressive pathotypes to evolve through the recombination of distinct pathotypes and rapidly spread^18^. In rye, the causative agent of PM, *B. graminis* f. sp. *secalis*, has attracted little scientific interest in recent years^19,20^.

To reduce our dependency on pesticides, host resistance constitutes a sustainable alternative for farmers to ensure crop productivity^21^. At present, 23 major PM resistance (*R*) genes have been identified in rye (Table 1). However, it is likely that a subset of colocalized *R* genes are allelic, as there was no evidence that these genes were distinct from existing *R* genes prior to denotation.

**Table 1:** Location and origin of powdery mildew resistance genes in rye (*Secale cereale* L.) and wheat- (*Triticum aestivum* L.) rye translocation lines with rye being the donor parent. After^1–3.^.

Chromosome	Arm	Gene name		References
		Rye	Wheat	
1R	Short	*Pm1*	*Pm8*	^22^
		*Pm1*	*Pm17*	^23^
		*Pm1*	*PmCn17*	^24^
		*Pm*		^2^
		*Pm*		^25^
	Long	*PmSESY*		^26^
	ND	*Pm7*	*–*	^27^
2R	Long	*Pm2*	*–*	^28^
		*–*	*PmJZHM2RL*	^29^
		*Pm*	*–*	^30,31^
	ND	*–*	*Pm7*	^32^
		*Pm8*	*Pm3*	^31^
		*Pm*		^33^
3R	Short	*Pm3*	*–*	^34^
4R	Long	*Pm*	*–*	^35,36^
	ND	*Pm*	*–*	^37^
		*Pm6*	*–*	^34^
5R	Long	*Pm4*	*–*	^38^
6R	Short	*–*	*Pm56*	^39^
	Long	*–*	*Pm20*	^40^
		*Pm5*	*–*	^41^
		*Pm*	*–*	^31,36^
7R	Long	*Pm*	*–*	^42^

ND: Interchromosomal position not determined.

The majority of characterized *Pm* genes encode intracellular nucleotide-binding leucine-rich repeat (NLR) proteins that recognize pathogen effector molecules, leading to an effector-triggered immunity resistance response^43–45^. Canonical NLR genes are composed of three domains^46^. In grasses, the N-terminus is composed of a coiled-coil (CC) domain believed to be involved in signaling and the induction of cell death^47^. In the center, a nucleotide-binding adaptor shared by APAF-1, R proteins, and the CED-4 (NB-ARC) domain functions as a regulatory domain determining the protein activation state^48^. Last, in the C-terminus, a leucin-rich repeat (LRR) domain is involved in effector recognition^49^. In rye, 770 canonical NLR genes have been identified in the ‘Lo7’ reference genome^50^.

Rye has been an important source for the improvement of PM resistance in wheat (*Triticum aestivum* L.) by chromosomal translocation of segments housing *R* genes^3,25,42^. While PM is effectively controlled by host genetic resistance in cereals, it has historically been rapidly overcome by virulent races of *Bg*^51,52^. Currently, several of the top-yielding hybrid rye cultivars examined in official Danish trials are susceptible to PM disease (Supplementary Table S1)^53^.

Genomic-based breeding techniques have, however, dramatically accelerated the introgression and pyramiding of several *R* genes for enhanced resistance durability^54–56^. Recent advances in genomic resources available in rye, including the 600 K SNP array and chromosomal-scale reference genome of the German inbred winter rye line ‘Lo7’ and Chinese population rye variety ‘Weining’, constitute significant milestones in rye genomic breeding^50,57,58^. Continuous mining for the discovery of novel genetic variability in rye *R* genes is essential to expand the ‘toolset’ available for resistance breeding. The importance of genetic studies in rye is further supported by the possibility of introgressing novel *R* genes into the staple cereal wheat by chromosomal translocation lines^3^.

In this paper, we investigate PM resistance in a less-prevalent elite Gülzow-type hybrid rye breeding germplasm. Our objective was to I) characterize PM resistance in the assayed germplasm, II) identify PM resistance-associated SNP markers to be implemented by marker-assisted selection for breeding novel resistant hybrid rye cultivars, III) investigate whether NLR genes residing in PM resistance-associated blocks on the ‘Lo7’ and ‘Weining’ reference genomes resemble known *Pm* genes, and IV) develop a marker map for the 600 K high-density SNP array and validate its performance in the assayed germplasm.

## Results

### 600 K SNP genotyping of panel

**Figure 1:**
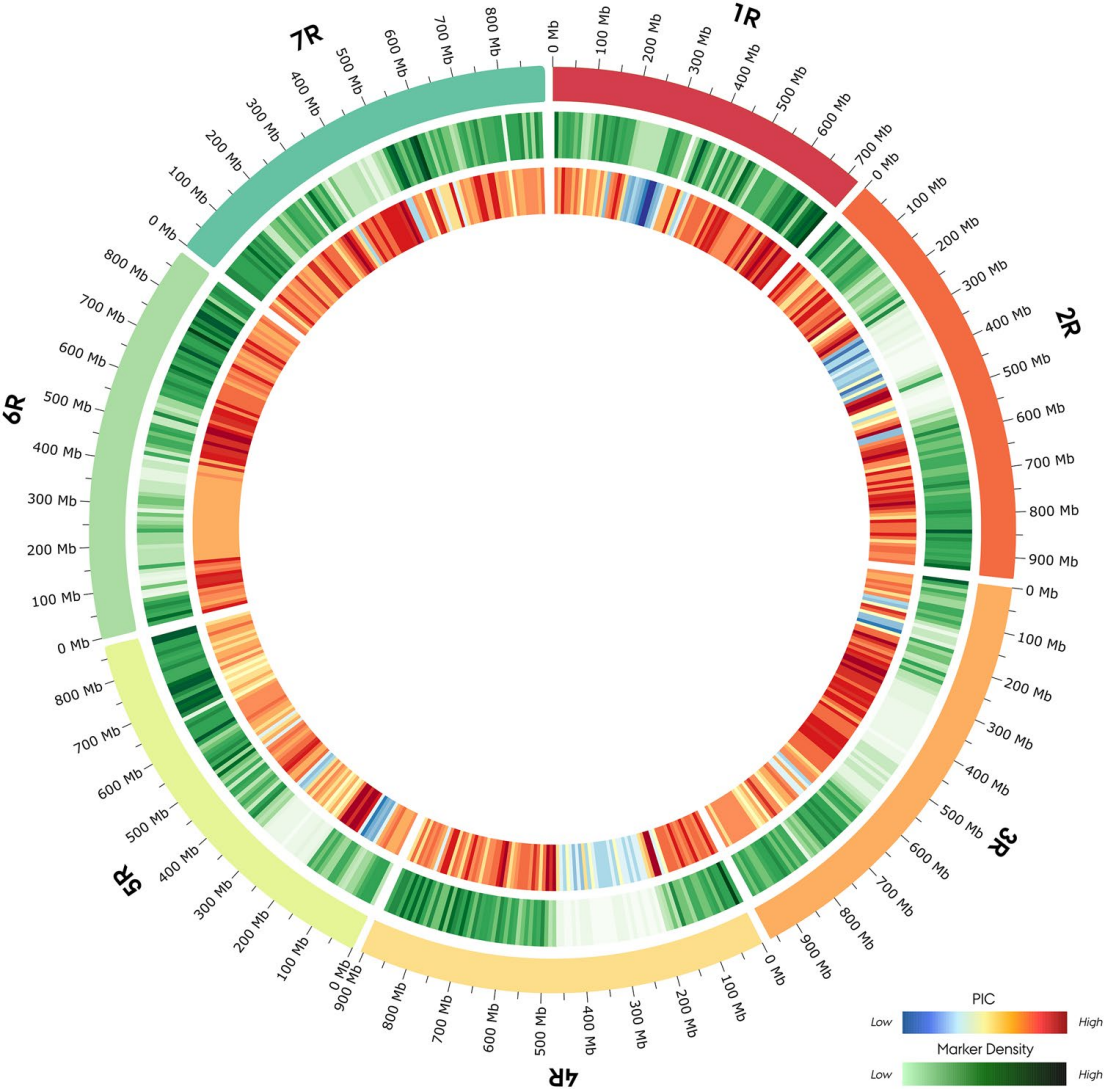
Distribution of 244,463 informative 600 K SNP array markers in 180 Nordic Seed hybrid rye (*Secale cereale* L.) breeding lines along the ‘Lo7’ reference genome in 10 mb bins. The outer track depicts the marker density per bin. The inner track depicts the polymorphism-information content (PIC) per bin.

To investigate the genetics underlying powdery mildew (PM) resistance, the assayed hybrid rye breeding germplasm was genotyped on the rye 600 K SNP array. With only scaffold positional data available for the array, SNP marker sequences were anchored to the recent ‘Lo7’ rye reference genome and stringently filtered to ensure its accuracy. In total, 591,196 markers were successfully mapped to the reference genome, and the developed marker map was made available at https://doi.org/10.5281/ZENODO.5086235. Quality filtration of markers for low minor allele frequency, missing markers, and missing individual scores across the panel led to the identification of 261,406 informative markers (Supplementary material 1). Characterization of fundamental performance-related metrics revealed a homogeneous inter- and intrachromosomal distribution of markers (Table 2). On average, each chromosome housed 32,676 markers with a mean marker-to-marker distance of 25.54 kb. The largest marker-to-marker distance was 9.95 Mbp on chromosome 2R, with a mean of 4.05 Mbp across the chromosomes. As a quality parameter, the polymorphism information content (PIC) was calculated to estimate the ability of markers to detect polymorphisms within the assayed germplasm (Supplementary Table S2). Across the informative marker panel, a mean PIC of 0.234 was identified, with a mean interchromosomal PIC ranging from 0.204 to 0.249 (Table 2). Visualization of these array performance metrics along the rye genome using Circos revealed a drop in marker density and PIC across the pericentromeric region on all chromosomes (Fig. 1).

**Table 2:** Characteristics of informative 600 K SNP array on the Nordic Seed hybrid rye (*Secale cereale* L.) elite breeding germplasm (n = 180). Markers were positioned on the ‘Lo7’ reference genome.

Chromosome	Chromosome length (Mbp)	Markers	Informative SNP markers			
			Markers	Mean inter- marker distance ± SD (kb)	Largest inter- marker distance (Mbp)	PIC ± SD
1R	727.33	72,089	33,854	21.47 ± 54.7	2.43	0.204 ± 0.134
2R	945.85	77,774	33,698	28.07 ± 96.5	9.95	0.240 ± 0.120
3R	965.54	69,428	31,493	30.65 ± 75.4	3.60	0.238 ± 0.111
4R	906.54	81,652	32,555	27.84 ± 75.5	2.98	0.230 ± 0.125
5R	876.06	81,842	37,073	23.63 ± 66.9	3.78	0.238 ± 0.123
6R	885.15	84,283	36,872	24.00 ± 62.2	3.31	0.249 ± 0.105
7R	889.76	86,994	38,918	23.12 ± 56.6	2.31	0.231 ± 0.120
Unmapped	–	35,245	16,943	–	–	0.239 ± 0.123
Mean	886.59	74,869	32,676	25,54 ± 69.7	4.05	0.234 ± 0.121

SD: Standard deviation, PIC: Polymorphism information content.

### Phenotyping of hybrid rye breeding germplasm

To provide a comprehensive phenotypic dataset of PM resistance in the assayed Gülzow-type hybrid rye breeding germplasm, the lines were scored for their infection type (IT) against three distinct *Blumeria graminis* f. sp. *secalis* (*Bgs*) populations (Table 3, Supplementary Table S3). The lines scoring an IT below 1 were considered ‘resistant’ (Supplementary Fig. S1). Across the assayed germplasm, the N13 (Nienstädt, 2013) *Bgs* population yielded a mean IT of 2.40 ± 1.28 standard deviations (SD), with 47 resistant lines out of which 3 were restorers. The N18 (Nienstädt, 2018) *Bgs* population yielded a mean IT of 2.71 ± 1.11 SD with 29 resistant lines, out of which 1 was a restorer. D20 (Dyngby, 2020) *Bgs* population yielded a mean IT of 2.74 ± 1.10 SD with 20 resistant lines out of which 1 was a restorer. Across the assayed germplasms, 20 out of 88 non-restorer germplasm and 1 out of 92 restorer lines were consistently resistant to all three *Bgs* populations. Both controls, hybrid cv. KWS Binntto (‘susceptible’) and KWS Serafino (‘resistant’) were susceptible to all three *Bgs* populations. KWS Binntto had a mean IT of 3.56 ± 0.54 SD and KWS Serafino 3.01 ± 1.01 SD across *Bgs* populations.

**Table 3:** Nine-step 0–4 scale for scoring infection types in cereal powdery mildew, after Torp et al.^88^.

Phenotype	Infection-type	Mycelium growth	Sporulation	Development of chlorosis/necrosis
Resistant	0	None	None	No
	0–1	None	None	Yes
	1	Weak	None	Yes
Partially resistant	1–2	Weak	Weak	Yes
	2	Moderate	Weak	Yes
Partially susceptible	2–3	Moderate	Moderate	Yes
	3	Strong	Moderate	Yes
Susceptible	3–4	Strong	Strong	Yes
	4	Strong	Strong	No

**Figure 2:**
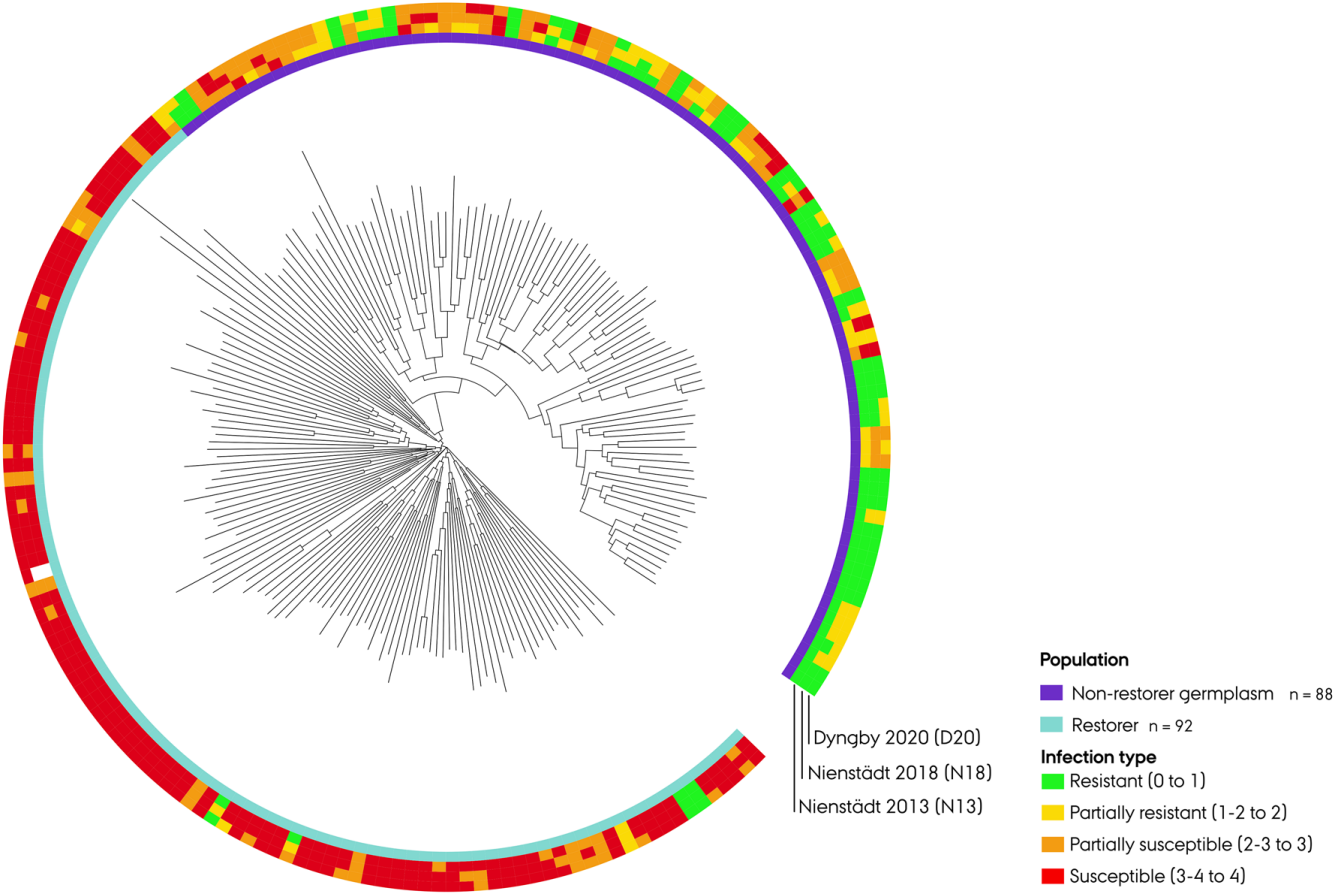
Circular neighbor-joining dendrogram of 180 hybrid rye (*Secale cereale* L.) breeding lines. Infection type (0–4) reaction against three powdery mildew populations displayed by concentric circles around the dendrogram.

To visualize the resistance spectrum of breeding lines, a circular neighbor-joining dendrogram was constructed, and concentric circles were added to integrate the scored IT (Fig. 2).

**Figure 3:**
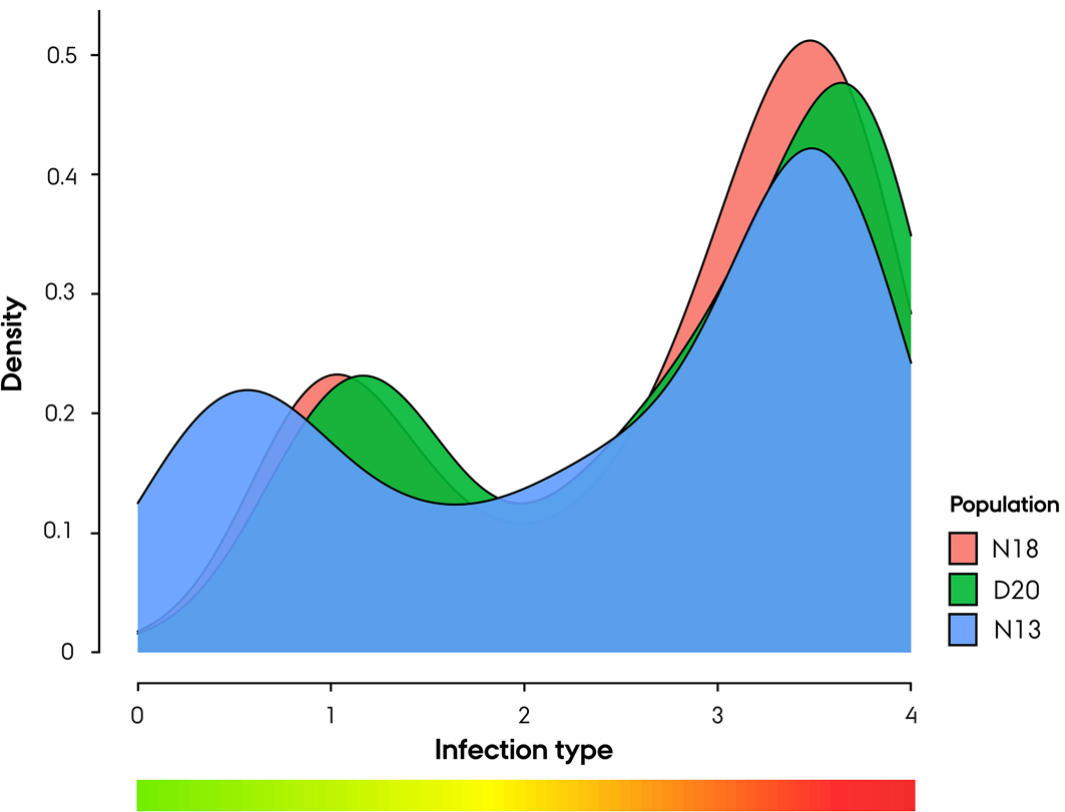
Density plot of the infection type distribution across 180 Nordic Seed hybrid rye (*Secale cereale* L.) breeding lines against three powdery mildew populations.

Statistical analysis of the line infection-type distribution across *Bgs* population by ANOVA showed that the N13 *Bgs* population differed significantly from the N18 and D20 *Bgs* populations (*p*_*val*_ < 0.00016) (Fig. 3). Nine non-restorer germplasm lines were found to exhibit a differential resistance response to the N13 *Bgs* population. These lines were categorized as ‘partially resistant’, with a mean infection type of 1.52 ± 0.58 SD; they exhibited a mean infection type of 2.74 ± 0.70 and 2.80 ± 0.77 against the N18 and D20 populations, respectively.

### Genome-wide association study

**Figure 4:**
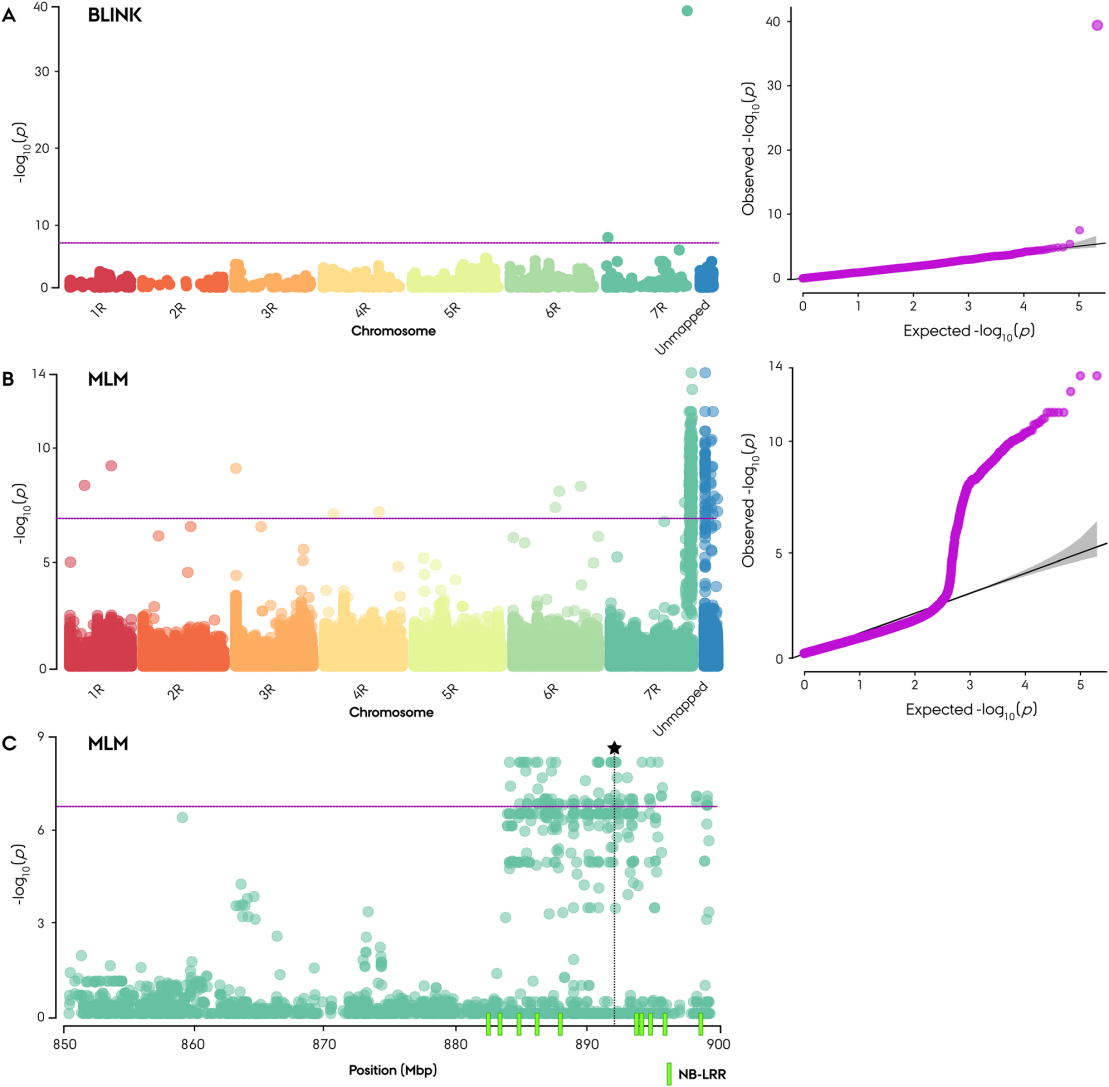
Manhattan plot for the genome-wide association study (GWAS) of powdery mildew disease resistance in Nordic Seed hybrid rye (*Secale cereale* L.) elite breeding germplasm (n = 180) using 261,406 informative SNP markers. (**A**) GWAS using BLINK including a Q–Q plot. (**B**) GWAS using the MLM method including a Q–Q plot. (**C**) GWAS using the MLM method for the PM resistance-associated region on chromosome arm 7RL. Asterisks denote the position of the top-associated SNP marker identified in BLINK GWAS. NLR genes on the ‘Lo7’ reference genome are represented by green bars. The purple line represents the Bonferroni adjusted significance threshold based on informative markers.

For the identification of SNP markers associated with PM resistance, GWAS using MLM and the BLINK method was used for each of the three *Bgs* population trials and across trials for the entire germplasm and individual parental populations (Supplementary Figs. S2, S3). Isolated single SNPs in GWAS-MLM results were removed from the analysis even if they were significantly associated, as these were interpreted as having spurious associations or incorrect mapping positions. Instead, dense peaks comprising a large number of significant associated markers within a confined region in GWAS-MLM were selected for further analysis. GWAS-MLM on the entire panel led to the identification of a haplotype block on chromosome arm 7RL that was significantly associated (− log_10_ = 14.6) with powdery mildew resistance spanning from 882 to 898 Mbp (Fig. 4B, C). The haplotype block harbored 244 markers exhibiting an association above the Bonferroni adjusted significance threshold (− log_10_ ≥ 6.7) (Supplementary Table S4. Successive GWAS-BLINK led to the identification of the top-most resistance-associated (− log_10_ = 37.9) marker within the haplotype block on chromosome arm 7RL at 892.09 Mbp, which explained 16.8% of the phenotypic variance (Fig. 4A).

In the non-restorer germplasm population, lines were found to carry a highly conserved haplotype across the significantly associated markers on chromosome arm 7RL (Supplementary Table S6). A resistant haplotype was conserved in 42 out of 48 resistant lines and a susceptible haplotype was found in 28 out of 31 susceptible lines, and the remaining 9 lines showed a differential resistance response (Supplementary material S3).

GWAS using BLINK led to the disappearance of the marker position on the ‘Unmapped’ chromosome that was in linkage disequilibrium with the genomic region on chromosome arm 7RL (Fig. 4A, B). In the non-restorer germplasm, an additional nonsignificant (− log_10_ = 3.62) peak was identified in GWAS-MLM spanning from 743.9 to 754.4 Mbp on chromosome arm 5RL (Supplementary Fig. S2, Supplementary Table S4). Gene mining by GWAS in the restorer population alone was not performed due to the low number of resistant lines (n = 5).

### Nucleotide-binding leucine-rich repeat proteins in powdery mildew resistance-associated blocks on chromosome arm 7RL

Annotation of NLR genes in the reference genomes ‘Lo7’ and ‘Weining’ led to the identification of 770 and 1027 full-length (‘complete’) NLR genes, respectively. Positional information, annotation, and NB-ARC and NLR sequences of all ‘complete’ and ‘partial’ NLR genes for both reference genomes have been made available at https://doi.org/10.5281/zenodo.5085854. The PM resistance-associated block residing in the subtelomeric region of chromosome arm 7RL spanned 17 Mbp on the ‘Lo7’ reference genome and harbored 25 NLR genes (Fig. 4C, Supplementary Table S8). Out of the 244 markers residing in the block significantly associated (− log_10_(*p*) > 6.72) with PM resistance, 155 were accurately positioned on the ‘Weining’ reference genome (Supplementary Table S7). The markers mapped to a site spanning 14 Mbp from 994.6 to 1,008.4 Mbp on chromosome arm 7RL and housed 16 NLR genes (Supplementary Table S8). A search in the protein database using the NCBI Blastx function showed that the majority of NLRs shared similarities with resistance gene analogs (RGAs) and *Pik-2-like* and *Pik6*-*NP*-like disease resistance proteins in the diploid parental species of wheat. In both reference genomes, two NLRs shared similarities with *Rpp13-like* disease resistance proteins in *Triticum urartu* and barley.

**Figure 5:**
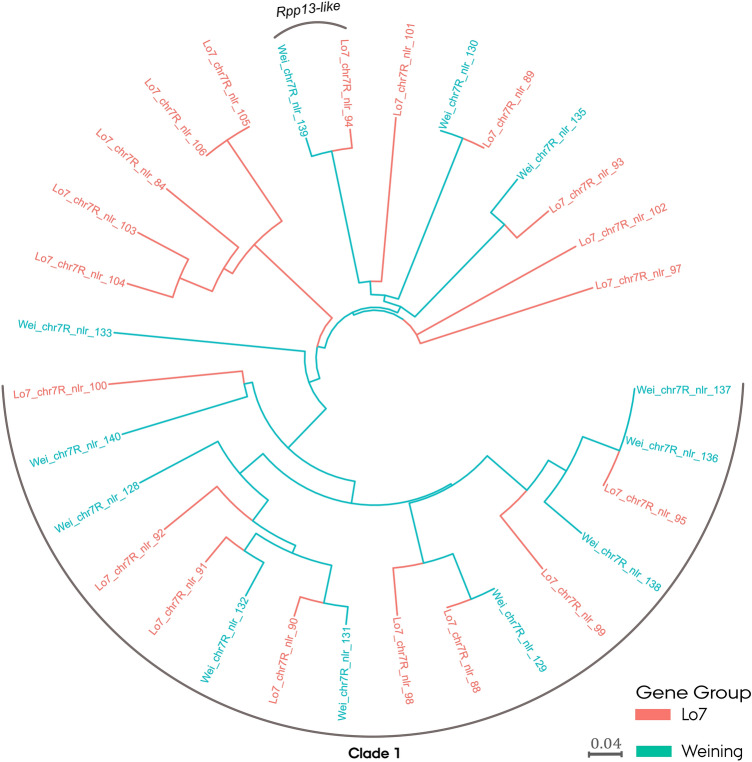
Phylogenetic tree of nucleotide-binding leucine-rich repeat (NLR) protein NB-ARC domains in the ‘Weining’ and ‘Lo7’ rye (*Secale cereale* L.) reference genomes. NLR genes are located at a powdery mildew resistance-associated site in the subtelomeric region of chromosome arm 7RL.

Phylogenetic analysis using the NB-ARC domain of NLRs residing in the PM resistance-associated haplotype block on chromosome arm 7RL led to the finding that the majority of the NLRs were represented in both reference genomes (Fig. 5). The ‘Weining’ reference genome exhibited one unique NLR not present in ‘Lo7’, while the latter exhibited eight unique NLRs, of which five formed a distinct clade. Eight homogeneously represented NLRs from each reference genome clustered in a large clade (‘clade 1’).

**Figure 6:**
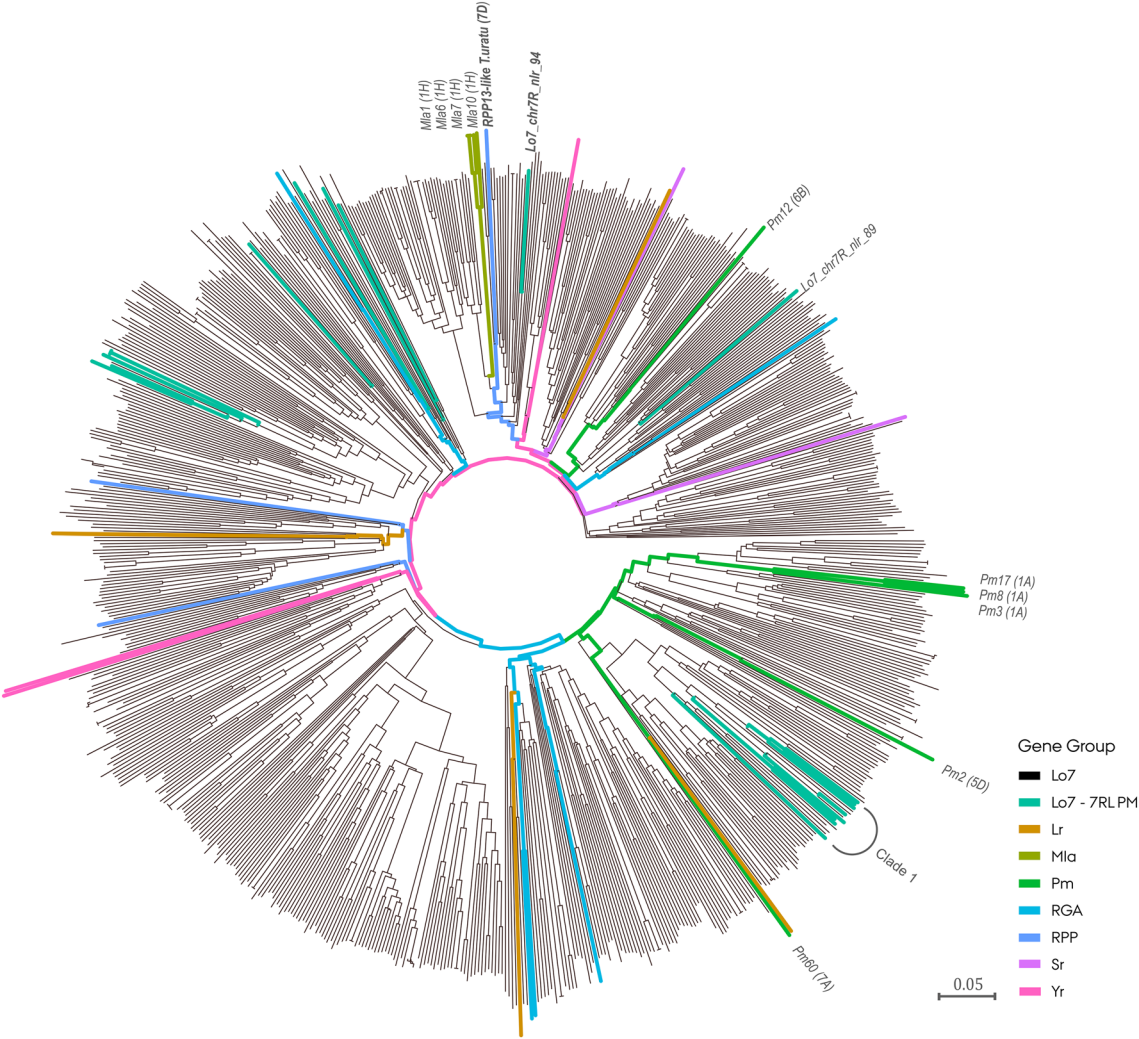
Phylogenetic tree of 770 nucleotide-binding leucine-rich repeat (NLR) protein NB-ARC domains in the ‘Lo7’ rye (*Secale cereale* L.) reference genome. The NB-ARC domains of known NLR genes have been included as references. NLR genes residing in a powdery mildew resistance block in the subtelomeric region of chromosome arm 7RL (Lo7–7RL PM) are colored teal.

An additional phylogenetic analysis was performed using the entire reference genome NLR repertoire including NLR genes of characterized *R* genes as references (Fig. 6, Supplementary Fig. S4). The NLR genes residing within the haplotype block were found to span much of the reference NLR repertoire diversity. Clade 1 remained intact in both reference genome NLR repertoire trees positioned in a section harboring four out of five reference *Pm* genes included, with the closest being *Pm60*. In both reference genome NLR repertoire trees, a single NLR (Lo7_chr7R_nlr_94, Wei_chr7R_nlr_139) showed an evolutionary relationship with an *Rpp13*-like disease resistance gene and four *Mla* alleles.

### Characterization of an *Rpp13*-like NLR gene residing in the resistance-associated block on chromosome arm 7RL

While several of the NLR genes residing within the PM resistance-associated haplotype block on chromosome arm 7RL chromosome showed evidence of an evolutionary relationship with known *Pm* genes, we selected the *Rpp13*-like NLR gene for further investigation on the basis of its close proximity. The *Rpp13*-like NLR gene (Lo7_chr7R_nlr_94, Wei_chr7R_nlr_139) was the closest NLR gene, residing approximately 2 Mbp from the top-most PM resistance-associated marker identified by BLINK (Fig. 4C, Supplementary Table S8).

The homologous *Rpp13*-like NLR genes in the ‘Lo7’ and ‘Weining’ reference genomes encoded canonical NLR proteins of 922 to 947 aa, showing 97% sequence similarity, with sequences differing by three single amino acid variants and an indel of 25 aa in the NB-ARC domain (Supplementary material 2). Protein BLAST of the NLR gene led to 88% sequence similarity with disease resistance *Rpp13-like* protein 4 in *T. urartu*, which encodes a 924 aa NLR protein sequence.

## Discussion

In Denmark, the top-yielding hybrid rye cultivars and population varieties were evaluated in Danish official trials as an advisory service for farmers^53^. In the last decade, a high level of powdery mildew infection in rye was recorded only once in the trials in 2017 at a site in southern Denmark (Supplementary Fig. S1). At the trial site, several hybrid rye cultivars displayed up to 18% leaf area covered by PM. While no recent studies have investigated the effect of powdery mildew in top-yielding hybrid rye cultivars, Matzen et al. (2019) reported a 16% yield reduction in triticale at a disease severity of ≤ 10% leaf area covered by powdery mildew under field conditions in 2017. It is, therefore, reasonable to presume that under certain conducive conditions, PM is capable of causing substantial grain yield and quality losses in rye. Furthermore, on the basis of the trial records, we selected a hybrid cv. KWS Serafino as a resistant control in our study, as it showed less than 1% leaf area covered by PM at the site in 2017. However, under a high disease pressure, cv. KWS Serafino was found to be susceptible in the current study, suggesting a potential lower level of resistance against PM in the evaluated top-yielding hybrid cultivars than evident from the official trials.

In this study, we investigated PM resistance in the less-prevalent Gülzow-type elite hybrid rye breeding germplasm against three distinct *Blumeria graminis* f. sp. *secalis* (*Bgs*) populations from Denmark and Northern Germany. We observed a moderate level of powdery mildew resistance in the non-restorer germplasm population, and by performing a genome-wide association study (GWAS) using 261,406 informative SNP markers, we identified a strong PM resistance-associated site on the distal region of chromosome arm 7RL.

Hybrid rye breeding germplasms are highly secluded with little exchange of material. The existing exchange is subsequently influenced by the deployed fertility control system, which determines the compatibility of foreign material for introgression into heterotic parental populations^59^. Additionally, the 600 K SNP array was developed using lines from a German hybrid rye breeding germplasm deploying the predominant Pampa-type cytoplasmic male sterility system (CMS)^57^. In contrast, the germplasm assayed in this study deploys the less-prevalent Gülzow-type CMS system^60^. Due to the distinct nature of the different hybrid rye breeding germplasms, we investigated the performance of the 600 K SNP array on the Gülzow-type germplasm. With no physical position data available for the 600 K SNP markers, we developed a marker map by anchoring the marker sequences to the ‘Lo7’ reference genome. In brief, we found that the 600 K SNP array yielded a dense panel of informative SNP markers in the Gülzow-type germplasm with markers homogeneously distributed across the rye genome. The level of marker informativeness measured by the polymorphism information content (PIC) was largely comparable with observations made in maize (*Zea mays* L.). Here, high-density SNP genotyping of 544 diverse CIMMYT inbred lines yielded 362 K informative SNP markers with a mean PIC of 0.25^61^. In this study, however, we did observe a drop in marker informativeness and density across the pericentromeric region. Similar observations were made in the study by Rabanus-Wallace, et al.^50^, who reported a considerable reduction in both genetic diversity and gene density within the pericentromeric region of the inbred rye line ‘Lo7’. In barley, this region has furthermore been observed to display a 20-fold lower recombination rate^62^. Thus, we concluded that the 600 K SNP array performed satisfactorily on the Gülzow-type germplasm in relation to the fundamental characteristics investigated. The high-density array constitutes a milestone in rye genomic resources transitioning SNP-based genomic studies and replaces the previous rye 5 K SNP array by Haseneyer, et al.^57,63^.

GWAS on SNP genotype data has become an effective tool in genome-based plant breeding for the study of oligogenic traits often governed by a few genes with large effects, such as the major monogenic inherited resistance (*R*) genes^63^. The use of high-density SNP typing has allowed whole-genome scans for the identification of often small haplotype blocks significantly associated with resistance^64^. The creation of the 600 K SNP array and the chromosomal-scale reference genomes ‘Lo7’ and ‘Weining’ in rye has significantly changed the genomic toolbox available for mining novel resistance genes^50,57^. Using these recent advances in rye genomic resources, we successfully managed to identify a site on chromosome arm 7RL that was significantly associated with PM resistance. The high level of resolution provided by the dense marker panel revealed that the PM resistance-associated haplotype block spanned 17 Mbp on the distal tip of the chromosome arm 7RL subtelomeric region.

Until recently, no PM resistance gene had been identified on the rye 7R chromosome. However, during the development of translocation lines for wheat improvement using a local Chinese variety of rye ‘Baili’, Ren et al.^42^ discovered a PM-resistant 7BS:7RL translocation line. With the recipient wheat parent being susceptible, the PM resistance gene traced back to the rye donor chromosome arm 7RL. Resistance phenotyping of the translocation line demonstrated that it displayed a high level of PM resistance against prevalent *Bg. tritici* pathotypes in China, making it very promising for the development of novel PM-resistant wheat cultivars. Although a novel finding in rye, several PM resistance genes have been identified in wheat chromosomal segments syntenic to rye chromosome arm 7RL. During *Triticeae* speciation, a series of recurrent translocation events gave rise to major patterns of chromosomal rearrangements^65^. In rye, the distal region of chromosome arm 7RL, therefore, shows high homology with the wheat 2A/B/D chromosomes^58,66^. Currently, more than five *Pm* genes have been identified in wheat on the 2A/B/D chromosomes^67,68^.

With the *Pm* gene discovered by Ren, et al.^42^ originating from a forage-type population of rye in a gene pool distinct from the germplasm investigated in this study, it is reasonable to presume that the two *Pm* genes could be either distinct or allelic^69^. We provisionally denote the novel *Pm* locus residing in the subtelomeric region of chromosome arm 7RL as *PmNOS1*.

Comparative analysis of the three *Bgs* populations used in the study revealed that the N13 population significantly differed from the two more recent populations, showing a less virulent composition of pathotypes. In addition, nine non-restorer germplasm breeding lines displaying a differential resistance profile were identified, showing ‘partial resistance’ only to the N13 population. With none of these lines carrying the resistance haplotype associated with the resistance locus on chromosome arm 7RL, our findings suggest that these do not carry the *PmNOS1* locus. While this observation can be explained by the occurrence of recombination events between the PM resistance-associated marker and the causative gene, this result seems less likely due to the consistent divergence in the haplotype^70^. Instead, it seems more likely that the differentially resistant non-restorer germplasm lines carry a distinct *Pm* gene that lost its effect during the period 2013–2018 in northern Germany. As a result of host genetic uniformity and the high evolutionary capacity of *Bg* to acquire virulence and migrate rapidly over long distances, the effectiveness of *Pm* genes is often rapidly lost. An example of this is the *Mla13 Pm* gene introduced in a former Czechoslovakian barley cultivar, ‘Koral’, in 1980^17^. After years of providing effective resistance against PM disease, virulence was observed in England in 1988 in a pathotype believed to originate from Czechoslovakia, having migrated by wind across the European continent and North Sea^71^. Ongoing monitoring of the virulence structure of *Bg* was conducted to survey the effectiveness of deployed *Pm* genes in elite cultivars^72,73^. While the *Pm* gene present in the differentially resistant non-restorer germplasm lines has seemingly been overcome, our findings suggest that *PmNOS1* remains effective.

Enabled by recent advances in rye genomic resources, we investigated whether any of the NLR genes residing in the region harboring the *PmNOS1* locus resembled known *Pm* genes. In several crop species, including rye, NLR genes have been observed to accumulate at recombination hotspots in subtelomeric chromosomal regions^50,74,75^. Additionally, we identified several large clusters of NLR genes residing in the PM resistance-associated block harboring *PmNOS1* in both of the reference genomes. In addition to the PM resistance gene identified in our study, Fusarium head blight and leaf rust resistance genes have been mapped to the subtelomeric region of chromosome arm 7RL^76,77^. Stem and stripe rust resistance genes reported in the 7BS:7RL translocation line developed by Ren et al.^42^ are furthermore likely to reside in the subtelomeric region. To investigate the likely diversity of *R* genes residing in the PM resistance-associated block, we conducted a phylogenetic analysis using the NB-ARC domain sequence of NLR genes residing in the block^78,79^. In contrast to the rapidly evolving LRR domain often exhibiting intraspecific polymorphism, the NB-ARC domain is largely conserved and suited for the study of evolutionary relationships among NLR genes^80,81^. As expected, the NLR genes residing in the block represented a large proportion of the NLR repertoire diversity in rye, accentuating the evolutionary plasticity of the NLRs residing in the subtelomeric region^75^. Intriguingly, guided by a panel of isolated NLR genes as a reference, we observed a predisposition of NLRs within the block to be in close proximity to known *Pm* genes in wheat and barley. This evolutionary relationship could hint at a common attribute among the NLRs^79^.

Phylogenetic analysis led to the identification of an NLR gene with an evolutionary relationship and protein sequence similarity to *Rpp13*-like protein 4 in *T. urartu.* Based on its close proximity to the marker displaying the strongest association with the *PmNOS1* locus, the *Rpp13*-like NLR gene was selected for further characterization. In *A. thaliana*, *Rpp13* confers resistance against *Peronospora parasitica*, the causative agent of downy mildew disease^82^. Recent studies have, however, identified *Rpp13-like* NLR genes associated with powdery mildew resistance in cereals. In wheat, Liu et al.^83^ showed that silencing of the *Rpp13* homologous gene *TaRPP13-3* in resistant wheat cv. ‘Brock’ induced susceptibility to powdery mildew. In barley, Cheng et al.^84^ found that the expression of an *Rpp13*-like NLR gene was highly upregulated after inoculation with powdery mildew.

In rye, while the rate of decay has only been determined in a few genes related to frost response, which showed a rapid linkage decay^85^, these genes have been demonstrated to decay after 3.76 kb, on average, across the genome in a similar hybrid breeding germplasm in maize^61^. Due to the heterogenic nature of outcrossing species, their rate of decay is often rapid^86^. However, in a recent population study on assayed germplasm, non-restorer germplasm was found to exhibit relatively low genetic diversity, low effective population size and high linkage disequilibrium^59^. Consistent with their observations, we found a large conserved haplotype on chromosome arm 7RL harboring the *PmNOS1* locus^59^. The linkage decay in the non-restorer germplasm is likely considerably reduced by the low genetic diversity and effective population size, resulting in a similarly low frequency of effective recombination events. The large amount of linkage, while beneficial for trait discovery in GWAS at lower marker density, impedes the identification of a narrow and precise genomic region that may harbor the gene of interest, even at high marker resolution. In the case of the *PmNOS1* locus, the haplotype block on chromosome arm 7RL was found to span 17 Mbp, harboring between 17 and 25 potential candidate NLR genes in the ‘Lo7’ and ‘Weining’ reference genomes. For more accurate mapping of the *PmNOS1* locus, the development of multiparent mapping populations could be conducted, allowing several generations of potential effective recombination events in the region^87,88^. Identification of the causative gene could be performed by resistance gene enrichment sequencing (RenSeq) analysis followed by transformation of a susceptible non-restorer germplasm line to validate the gene^89^. This would in turn equally show whether the gene is present in the reference genomes and whether *PmNOS1* encodes an *Rpp13-*like NLR protein.

In conclusion, our study demonstrates the immediate value of recent advances in rye genomic resources for the mining of novel resistance genes. These resources now permit accurate identification of delimited resistance-associated haplotype blocks and scanning for trait-associated genes residing within. With pathogens such as *Bg* displaying a large evolutionary plasticity, shortening the process from identification of resistance-associated sites to isolation of the underlying *R* gene is important for the development of novel resistant cultivars. The relevance of studies in rye is accentuated by the possibility of introgressing novel *R* genes into the staple cereal wheat by chromosomal translocation lines. To aid further studies in the field, we have provided both a rye600K SNP array marker map anchored using the ‘Lo7’ reference genome and NLR repertoire information of the ‘Lo7’ and ‘Weining’ reference genomes in open-access data repositories.

## Materials and methods

### Plant material and DNA extraction

A panel of 180 inbred rye (*Secale cereale* L.) lines, 92 restorer and 88 non-restorer germplasm, belonging to the elite Gülzow-type hybrid rye breeding germplasm at Nordic Seed A/S (Dyngby, Denmark) were investigated. Population structure and information on the genetic characteristics of the germplasm were presented in a recent study by Vendelbo et al.^59^. The parental populations represent genetically secluded gene pools with restorer (paternal) lines carrying a dominant allele for the restoration of male fertility and non-restorer germplasm lines carrying a recessive allele and fertile cytoplasm, which is used to maintain cytoplasmic male sterile (maternal) lines. DNA extraction was performed using an adapted SDS-based method according to the USDA^90^ after Pallotta et al.^91^ on an equivalent of 75 mg of plant material collected from the coleoptiles and primary leaves of two seven-day-old seedlings per line. The DNA concentration and 260/280 nm ratio of the samples were measured using an Epoch™ microplate spectrophotometer (Biotek®), and evidence of fragmentation was obtained by size visualization on a 1.2% agarose gel.

### Molecular marker resource and SNP genotyping

Samples of each line containing 200 ng of high molecular weight gDNA with a ≥ 1.8 260/280 nm ratio were sent for single nucleotide polymorphism (SNP) genotyping at Eurofins Genomics Europe Genotyping (Aarhus, Denmark). Genotyping was performed using a 600 K SNP array with 600,843 SNP markers on an Affymetrix GeneTitan™ Scanner platform^57^.

### Collection and multiplication of *Blumeria graminis* f. sp. *secalis* populations

As there was no unified information on germplasm resistance to powdery mildew, three field populations of *Bgs* were sampled in Northern Germany and Denmark in the period from 2013 to 2020 to screen the lines against a broad range of *Bgs* pathotypes prevalent in Northern Europe. Two *Bgs* populations were collected at the Nordic Seed rye multiplication site in Germany in 2013 (N13) and 2018 (N18) (52.29254°N, E9.14896°E). Ten leaves exhibiting PM disease were carefully collected and rinsed in 0.5 L of water to release *Bgs* spores. Six pots containing 15–20 susceptible 12-day-old seedlings of the restorer line R277 were then inoculated by spraying a fine mist of the spore solution using an atomizer bottle. Pots were transferred to a separate climate chamber for each population and incubated at 18 °C with 12 h of light using 400 W high-pressure Phillips SON-T Agro lamps. Populations were continuously multiplied in an overlapping two-week cycle. Each week, the tray of 2-week-old seedlings was substituted with a tray of fresh 12-day-old seedlings. Inoculation was achieved by passive dissemination of spores from the ‘older’ tray to the tray with new plants through a steel-grid shelf.

An additional *Bgs* population was collected in autumn 2020 (D20) at Nordic Seed (Dyngby, Denmark) (55.94944°N, E10.25414°E) using a mixture of hybrid cvs. KWS Binntto, KWS Bono and KWS Florano. Pots with 15 to 20 seedlings of the susceptible mixture were placed outdoors 12 days after sowing (DAS) in August-October 2020. Plants were controlled regularly, and all leaves showing PM disease within the period were collected. Prior to inoculation, leaves were placed in Petri dishes with moist filter paper and set to sporulate at room temperature in light for four to eight hours. Then, a pot containing 15–20 seedlings of the susceptible mixture was inoculated at 12 DAS by horizontally stroking the collected leaves across the seedlings. Inoculated pots were sprayed with a fine mist of water, placed in a container with transparent lids to ensure 100% RH and incubated in the dark at 10–15 °C for 24 h. After incubation in the dark, the pots were transferred to an isolated greenhouse cabin and incubated under 16 h of daylight at 18–24 °C and 8 h of dark at 14–16 °C.

Multiplication of trial inoculum for the three *Bgs* populations was performed using the same procedure as described above. For each population, two trays containing 35 pots of the susceptible mixture were inoculated by brushing 3–4 highly infected pots inoculated 20 days earlier across the tray.

### Infection and scoring

All lines were phenotyped for the infection-type response to the *Bgs* populations. In each greenhouse trial, eight seeds per line were sown in a 28-hole tray using a completely randomized design with two repetitions for each of the two trial replicates. For each tray, a positive (‘susceptible’) control consisting of hybrid cv. KWS Binntto and negative (‘resistant’) control cv. KWS Serafino was included. At 14 DAS, trays were inoculated as described above by brushing 3–4 highly infected pots inoculated 20 days earlier across the tray. After 14 days of incubation, the lines were phenotyped by scoring the infection response on the first and second leaves for each of the eight seedlings per repetition in accordance with a 9-step 0–4 scale by Torp et al.^88^ (Table 3).

### Data analysis

Bioinformatic analysis of SNP marker data was performed with the R studio (v. 1.3.959) interface in R statistical software (v. 4.0.1) by applying various predesigned packages^92,93^.

### Mapping of 600 K SNP array markers to the ‘Lo7’ reference genome

Positional data of the 600 K SNP markers were obtained by mapping each of the 600,843 SNP marker sequences to the rye reference genome ‘Lo7’ using the NCBI blastn (v. 2.9.0 +) function^50,94^. The mapping positions of SNPs were hereafter stringently filtered for I) complete SNP sequence alignment and II) a maximum of 1 mismatch to ensure accurate positioning.

### Molecular markers and characterization of 600 K SNP array performance

Prior to analysis, markers were filtered for a marker allele frequency ≥ 0.005, missing individual score ≤ 0.2 and missing marker score ≤ 0.1. Fundamental characteristics of SNP marker informativeness, including polymorphism information content (PIC), were calculated using the SnpReady (v. 0.9.6) R package^95^. The interchromosomal distribution of the informative marker PIC, marker-to-marker distance and marker density in 10 mb bins on the ‘Lo7’ rye genome were visualized using Circos (v. 0.69.8) in the Galaxy online interface^96,97^. A Circos plot was constructed using the pipeline developed by Hiltemann, et al.^98^.

### Analysis of phenotypic data

The distribution of infection types against the three respective PM populations was visualized by density plots using ggplot2 (v. 3.3.3) R package^99^. To determine whether the population infection-type distribution differed significantly, ANOVA was conducted using R.

To correct the resistance phenotype for the effects of replication and population, we fitted the data to a linear mixed model using the lme4 (v. 1.1.26) package in R:

$$y = \mu + P + R + l + \varepsilon$$

where µ is the general mean, P is the population, R represents the replications, *l* is the line id, and ɛ is the residuals. P and R were set as fixed effects, and *l* was set as a random effect. The random effect and residuals were assumed to be independent normally distributed variables described as follows: l ~ N (0, I ơ^2^_*l*_), and *ɛ* ~ N (0, I ơ^2^*ɛ*). The BLUP solutions for the line effect were used in GWAS. Data were also fitted to a linear mixed model for each of the populations to correct for the effect of replication. In this model ‘P’ was removed.

### Genome-wide association study

Discovery of PM resistance-associated SNP markers was performed by a genome-wide association study (GWAS) using the genomic association and prediction integration tool (GAPIT) (v.3) package in R^100^. The Manhattan plot was colorized using the RColorBrewer (v.1.1–2) R package color palette^99^. GWAS using a mixed linear model (MLM) was performed to identify discrete haplotype blocks associated with powdery mildew resistance. Additionally, the Bayesian-information and linkage-disequilibrium iteratively nested keyway (BLINK) method was performed to identify the top-most powdery mildew resistance-associated marker within each haplotype block^101^. BLINK uses a multiple loci test for MLM by combining a fixed effects model, Bayesian information content and linkage disequilibrium information to collectively improve the statistical power while simultaneously reducing the computational run time. Markers that are in linkage disequilibrium with the top-most significant marker at a site are excluded in BLINK. A standard Bonferroni-corrected threshold of α = 0.05 was used as the significance threshold.

#### Annotation of nucleotide-binding leucine-rich repeat proteins in the reference genome

To investigate whether PM-associated sites in the ‘Lo7’ and ‘Weining’ reference genomes housed nucleotide-binding leucine-rich repeat (NLR) genes, annotation was performed using NLR-parser (v.3) and NLR-annotator (https://github.com/steuernb/NLR-Annotator)^79,102^.

#### Characterization of nucleotide-binding leucine-rich repeat proteins

The gene structure, coding sequence and NLR protein sequence of candidate genes were extracted from RNA-seq and de novo protein data provided in ‘Weining’ and ‘Lo7’ reference genome data repositories^50,58^. To investigate whether NLR genes residing in PM resistance-associated sites resembled known genes, the NCBI blastx function was used for protein–protein searches in the online database^94^. For functional analysis and the prediction of protein domains, InterPro Scan was used^103^. Identification of sequence divergence between reference genome homologs was performed by multiple sequence alignment using the multiple sequence comparison by log-expectation (MUSCLE) method for coding sequences and the ‘Clustal Omega’ method for NLR protein sequences in Geneious Prime (v. 2020.2.3).

#### Phylogenetic analysis

Neighbor-joining clustering analysis of breeding lines was performed with Euclidean genetic distance measurement using the ape (v. 5.3) R package^104^. The tree was constructed after 10,000 bootstrapping iterations with weak nodes (≤ 80% recurrence) collapsed into multifurcations. A circular neighbor-joining tree was generated using the iTOL (v. 5) online tool (http://itol.embl.de/), enabling a colorful visualization of each line infection type spectrum against the three *Bgs* populations^105^.

To investigate the sequence similarity between nucleotide-binding leucine-rich repeat (NLR) genes at PM resistance-associated sites in the ‘Lo7’ and ‘Weining’ reference genomes, neighbor-joining clustering analysis using NB-ARC (nucleotide-binding adaptor shared by APAF-1, R proteins, and CED-4) domain sequences was performed. Identification of PM resistance-associated sites on ‘Weining’ was performed by mapping PM resistance-associated markers using the same procedure as described previously for ‘Lo7’. As references, NB-ARC domain sequences of available leaf rust (*Lr1, Lr10, Lr21, Lr22a*), stem rust (*Sr13, Sr22*), yellow rust (*Yr5, Yr10, Yr28*), powdery mildew (*Pm2, Pm3, Pm8, Pm12, Pm17, Pm60*, *Mla1, Mla6, Mla7, Mla10, RPP13, RPP-like-T. urartu*) and resistance gene analogs (*RGA1, RGA2, RGA3, RGA4, RGA5*) were included. NB-ARC sequences were obtained from the UniProt online database^106^. Phylogenetic analysis was conducted using a pipeline developed by Toparslan, et al.^107^ in R. Multiple sequence alignment of NB-ARC domain sequences was performed via msa (v. 1.20.1) using the ‘Clustal Omeage’ method and pairwise genetic distance based on identity calculated with the seqinr (4.2–8) package in R^108,109^. A tree was constructed for the ‘Lo7’ and ‘Weining’ NLR repertoires separately and visualized using ggtree (v. 2.2.4) R package^110^.

### Graphical editing

Graphs and figures were outputted from R in .svg format and manually curated using Inkscape (v. 1.1) (https://inkscape.org/).

### Ethical statement

Experimental research and field studies on plants (either cultivated or wild), including the collection of plant material,complies with relevant institutional, national, and international guidelines and legislation.

## Supplementary Information

Supplementary Table S1.

Supplementary Table S2.

Supplementary Table S3.

Supplementary Table S4.

Supplementary Table S5.

Supplementary Table S6.

Supplementary Table S7.

Supplementary Table S8.

Supplementary Legends.

Supplementary Information 1.

Supplementary Information 2.

Supplementary Figure S1.

Supplementary Figure S2.

Supplementary Figure S3.

Supplementary Figure S4.
