# Supplementary material for: Discovery of a novel powdery mildew (Blumeria graminis) resistance locus in rye (Secale cereale L.)
Source: Sci Rep. 2021 Nov 29;11:23057. doi: 10.1038/s41598-021-02488-5 (PMC8630102; doi:10.1038/s41598-021-02488-5)
Supplement: Supplementary file 9 — Supplementary Legends. [file 41598_2021_2488_MOESM9_ESM.docx]

**Supplementary Legends**

Supplementary material 1: Genotype of 261,406 informative 600K SNP markers in the Nordic Seed hybrid rye elite breeding germplasm (n=180) (.Rdata).

Supplementary material 2: Coding and protein sequences of the *Rpp13*-like NLR gene in the ‘Lo7’ (Lo7_chr7R_nlr_94) and ‘Weining’ (Wei_chr7R_nlr_139) rye (*Secale cereale* L.) reference genomes (.fasta) residing in close proximity to the top-most powdery mildew resistance-associated marker.

Supplementary Figure S1: Two inbred rye (*Secale cereale* L.) lines belonging to the Nordig Seed A/S hybrid rye breeding germplasm displaying complete susceptibility (left) and resistant (right) at high powdery mildew disease pressure in a greenhouse trial.

Supplementary Figure S2: Manhattan plot for genome wide association study (GWAS) using MLM method of powdery mildew disease resistance in an entire hybrid rye (*Secale cereale* L.) breeding germplasm (‘All’), or parental populations, restorer (‘R’, n = 92) and non-restorer germplasm (‘NRG’, n= 88) using 261,406 informative SNP markers. Lines were phenotyped using three distinct field populations from northern Germany (N13, N18) and Denmark (D20). The purple line represents the Bonferroni adjusted significance threshold based on informative markers

Supplementary Figure S3: Manhattan plot for genome wide association study (GWAS) using BLINK method of powdery mildew disease resistance in an entire hybrid rye (*Secale cereale* L.) breeding germplasm (‘All’), or parental populations, restorer (‘R’, n = 92) and non-restorer germplasm (‘NRG’, n= 88) using 261,406 informative SNP markers. Lines were phenotyped using three distinct field populations from northern Germany (N13, N18) and Denmark (D20). The purple line represents the Bonferroni adjusted significance threshold based on informative markers

Supplementary Figure S4: Phylogenetic tree of 1027 nucleotide-binding leucine-rich repeat (NLR) protein NB-ARC domains in the ‘Weining’ rye (*Secale cereale* L.) reference genome. The NB-ARC domains of known NLR genes have been included as references. NLR genes residing in a powdery mildew resistance block in the subtelomeric region of chromosome arm 7RL (Lo7 – 7RL PM) are colored teal.
