# Supplementary figures and images for: Discovery of a novel powdery mildew (Blumeria graminis) resistance locus in rye (Secale cereale L.)
Source: Sci Rep. 2021 Nov 29;11:23057. doi: 10.1038/s41598-021-02488-5 (PMC8630102; doi:10.1038/s41598-021-02488-5)

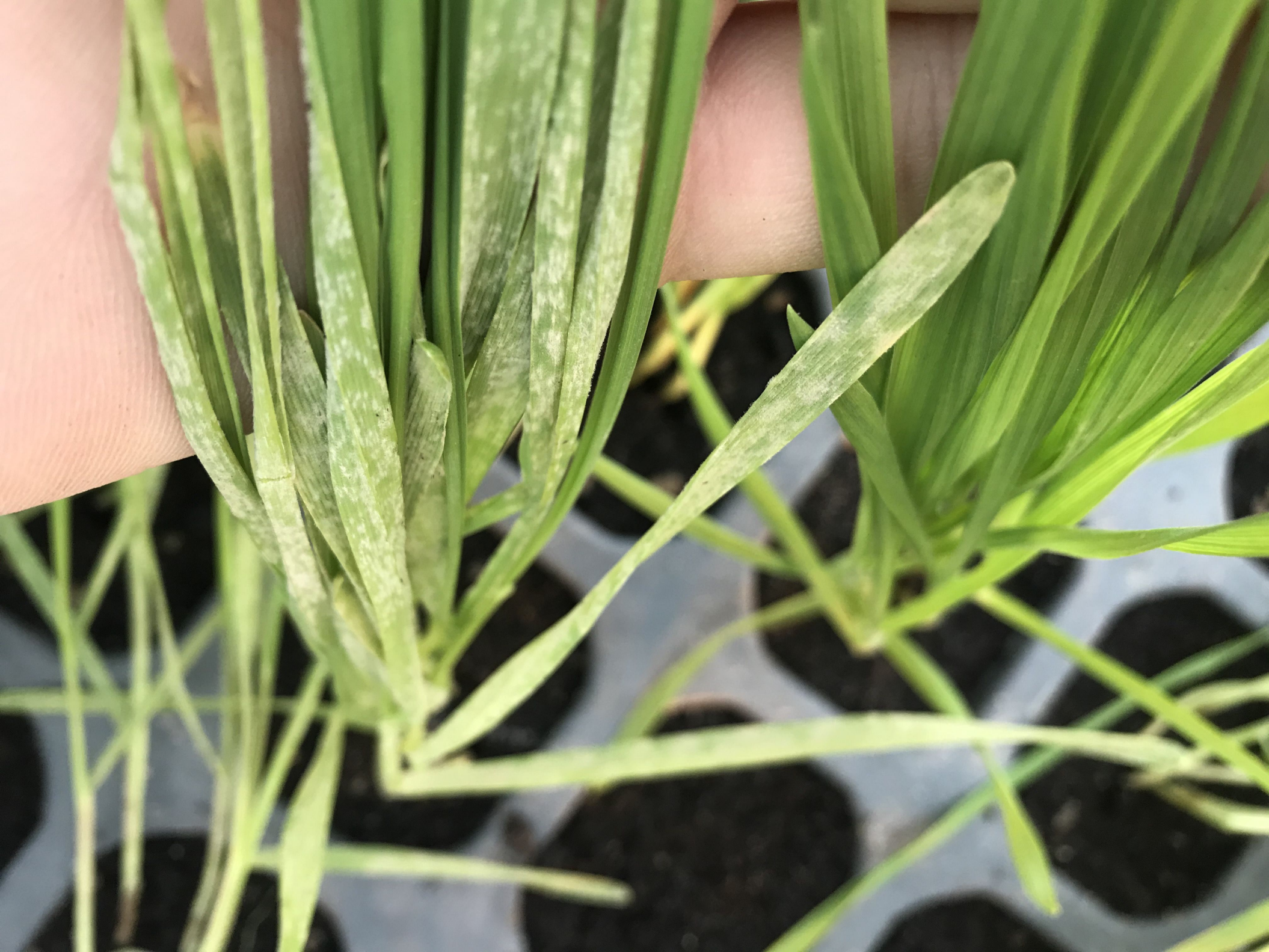

Supplement: Supplementary file 12 — Supplementary Figure S1. [file 41598_2021_2488_MOESM12_ESM.pdf]

**All**  
(n = 180)

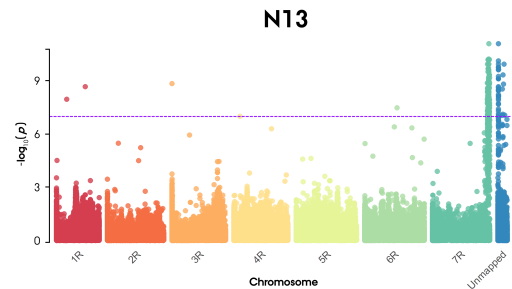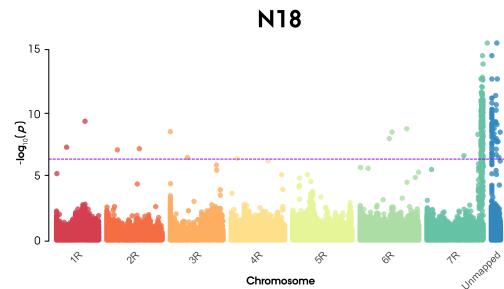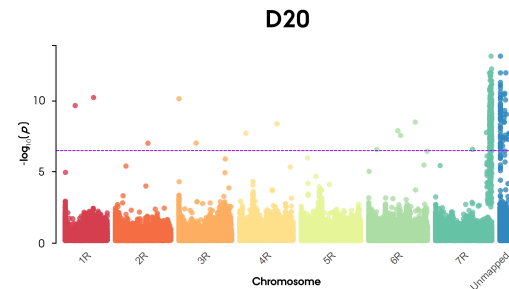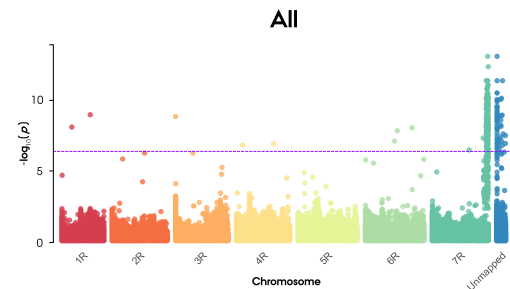

**NRG**  
(n = 88)

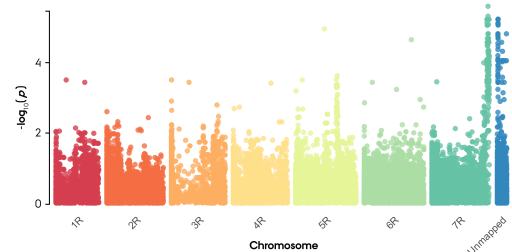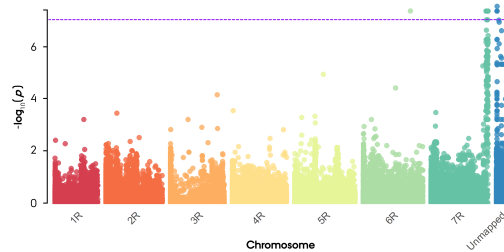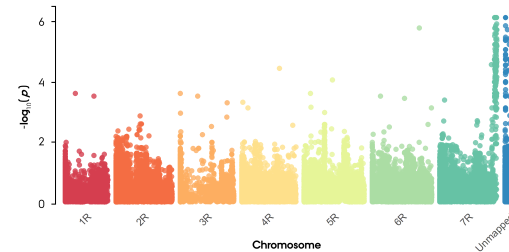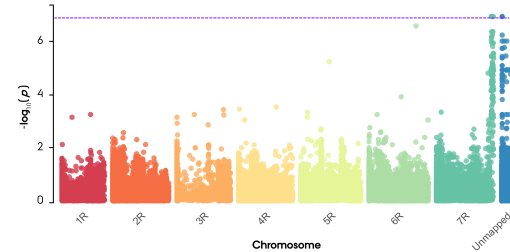

**R**  
(n = 92)

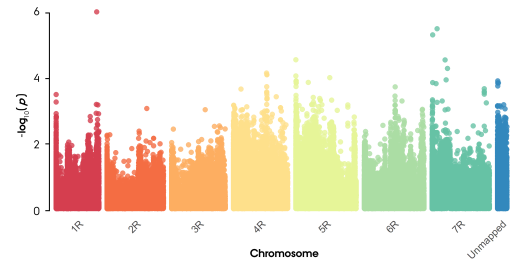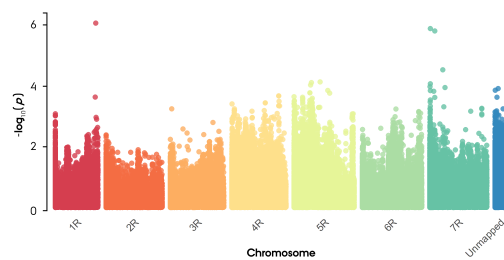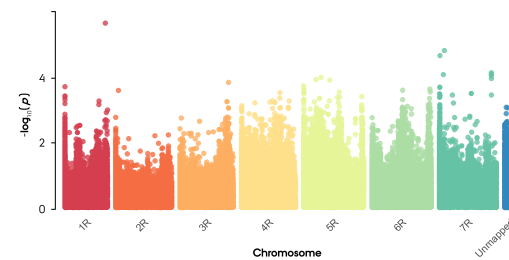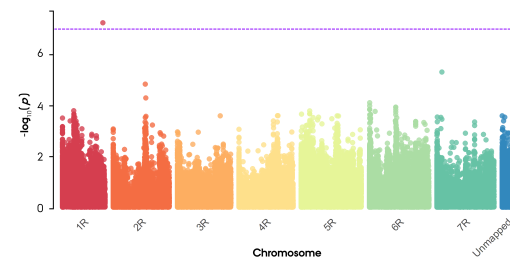

Supplement: Supplementary file 13 — Supplementary Figure S2. [file 41598_2021_2488_MOESM13_ESM.pdf]

**All**  
(n = 180)

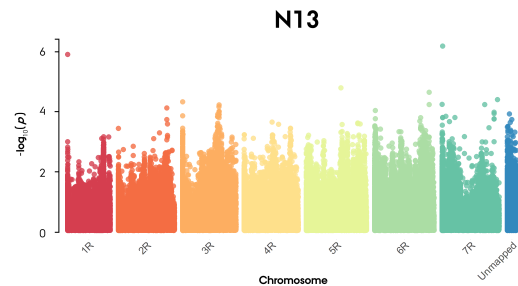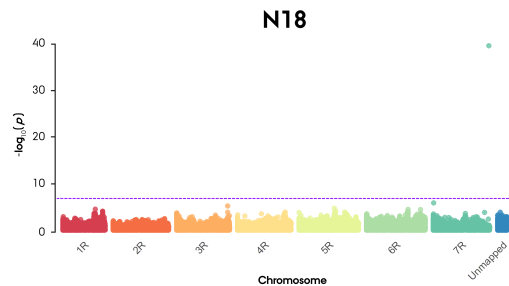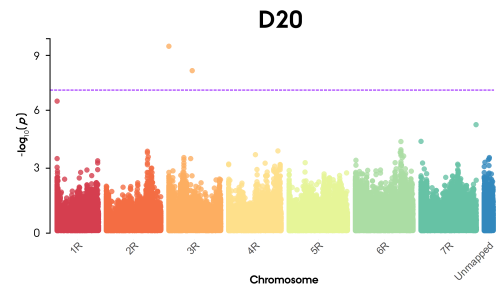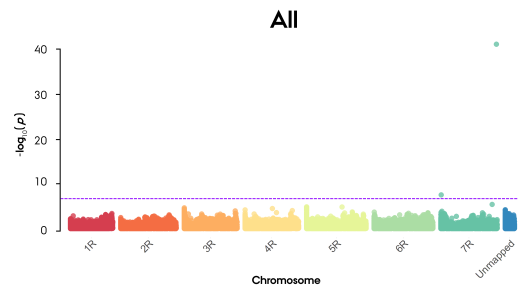

**NRG**  
(n = 88)

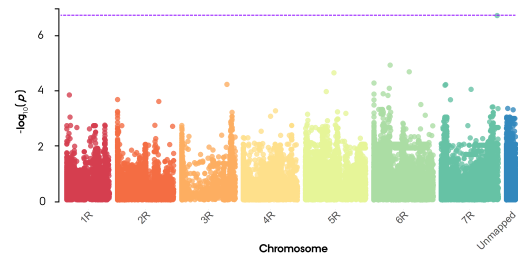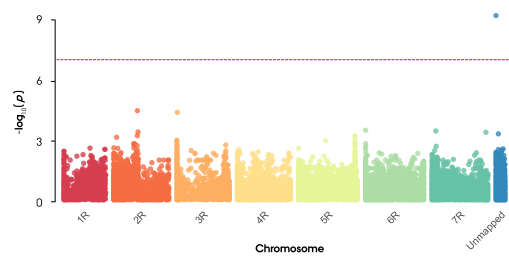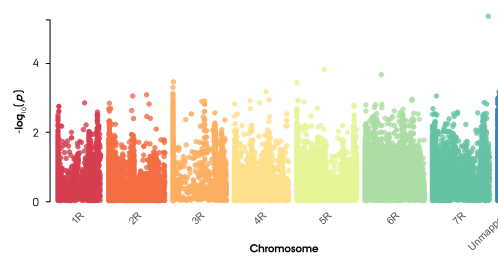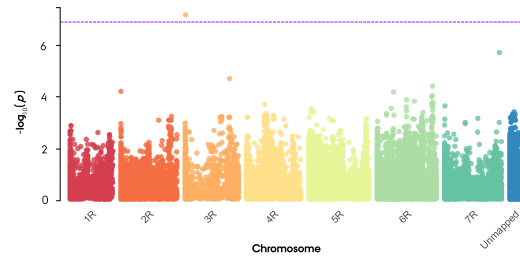

**R**  
(n = 92)

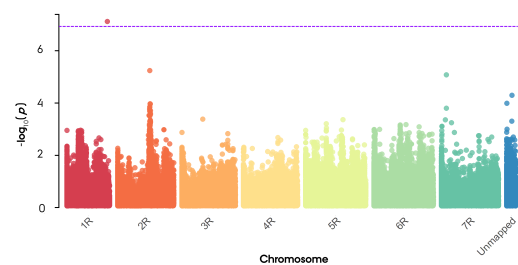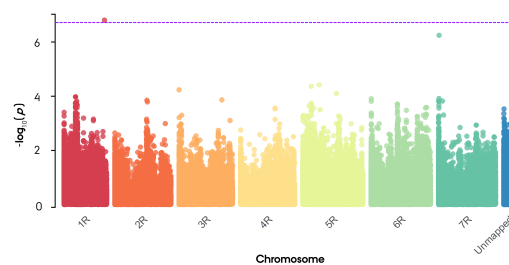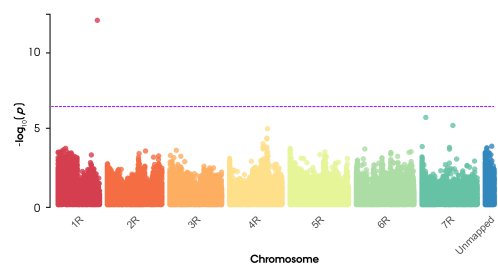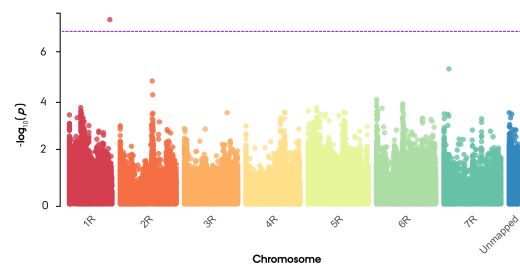

Supplement: Supplementary file 14 — Supplementary Figure S3. [file 41598_2021_2488_MOESM14_ESM.pdf]

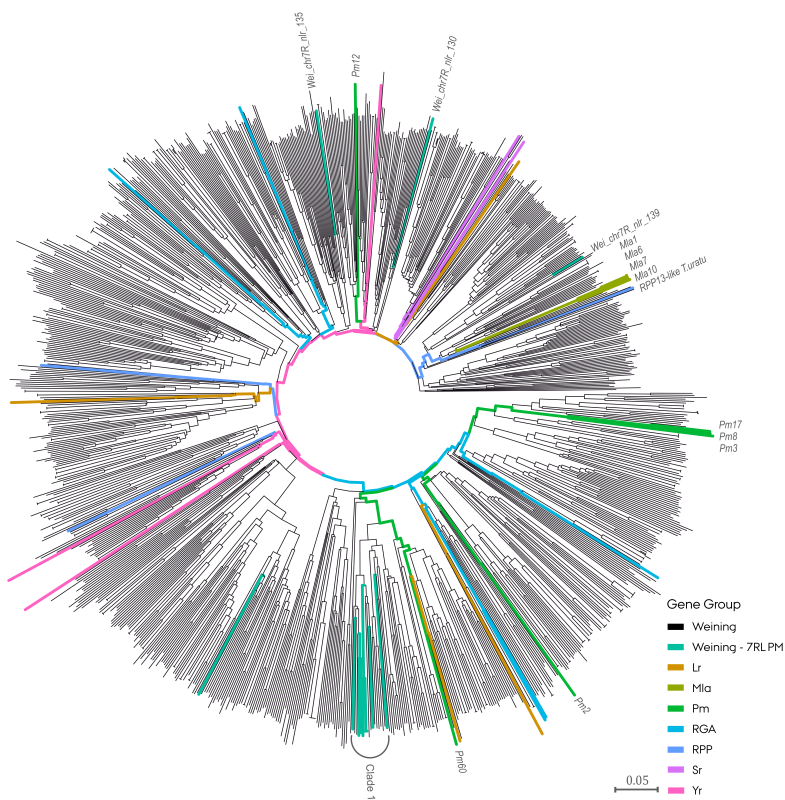

Supplement: Supplementary file 15 — Supplementary Figure S4. [file 41598_2021_2488_MOESM15_ESM.pdf]
